# Compression brace for secondary pectus carinatum in infants and toddlers undergoing cardiac surgery with midline sternotomy

**DOI:** 10.1007/s11748-024-02030-0

**Published:** 2024-04-25

**Authors:** Hayato Konishi, Kenta Fujiwara, Sayaka Okazaki, Akiyo Suzuki, Tatsuya Suzuki, Takahiro Katsumata, Toru Nagano, Shintaro Nemoto

**Affiliations:** 1 Department of Thoracic and Cardiovascular Surgery, Osaka Medical and Pharmaceutical University, 2-7 Daigaku-machi, Takatsuki, Osaka 569-0801 Japan; 2 Doi Orthopedic Clinic, Takatsuki, Osaka, Japan; 3 Nagano Prosthetics and Orthotics Co., Ltd., Moriguchi, Osaka, Japan

**Keywords:** Pectus carinatum, Midline sternotomy, Cardiac surgery, Compression brace

## Abstract

**Purposes:**

This study aimed to retrospectively assess the response to a newly developed compression brace for improving the deformity of the secondary pectus carinatum in infants and toddlers undergoing cardiac surgery with midline sternotomy. Factors affecting the response to the brace were identified.

**Methods:**

Fifty-one children were enrolled. Severity was expressed as the protrusion angle of the sternum obtained from chest X-ray. The patients were divided into two groups by positive or negative binary residuals of the relationship between the angle at the beginning and its percentage change after wearing the brace. Logistic regression analysis was used to identify the influencing factors.

**Results:**

Thirty patients (58.8%) showed zero and positive residuals to the relationship (good responders, Group G), whereas 21 patients showed negative residuals (poor responders, Group P). Male sex, severe cardiac anomaly, complex surgical procedure, multiple sternotomy, total duration, and self-discontinuation were associated with poor response to the brace by univariate analysis. The first three factors remained with high odds ratio for poor response by multivariate analysis. No adverse events occurred with the brace.

**Conclusion:**

Our newly developed compression brace contributed, at least in part, to improve the deformity of the secondary pectus carinatum. Further studies are required to clarify the therapeutic efficacy of anterior chest compression for secondary pectus carinatum.

**Supplementary Information:**

The online version contains supplementary material available at 10.1007/s11748-024-02030-0.

## Introduction

Anterior protrusion of the lower sternum may develop in infants and toddlers following cardiac surgery with midline sternotomy. This protrusion has not been a priority treatment target because it rarely causes significant symptoms. The protrusion can be referred to as secondary pectus carinatum (PC) and differs from primary PC in general, which has an unspecified genetic background and is often treated in adolescence for cosmetic or psychological reasons. The mechanism of secondary PC is unclear, but the protrusion is characterized by reactive tissue overgrowth of the parasternal cartilage caused by mechanical stimuli due to instability of the ribs and sternum during surgical manipulation [1, 2].

Various types of anterior compression braces have been widely used and have been shown to be effective for primary PC [3–8]. In contrast, there are no reports of the use of a compression brace in secondary PC developed after cardiac surgery in infants and toddlers. We have developed a newly designed brace dedicated to this population and introduced it in clinical practice since 2013. However, because the frequency, severity, and natural history of postoperative secondary PC have not yet been reported, we had no choice but to use our own severity assessment method and protocol to observe the deformity and the response to the brace.

In this retrospective study, the response to the compression brace in the secondary PC was assessed by measuring the percent change in the protrusion angle and the factors affecting the response.

## Methods

### Ethical statement

This retrospective study was approved by the Institutional Review Board of Osaka Medical and Pharmaceutical University (No. 2021-184) in compliance with the ethical guidelines for human life science and medical research based on the Declaration of Helsinki. Written informed consent was waived by the IRB, but oral consent forms from guardians were documented in electronic medical records before the start of installation.

### Development of a brace for secondary PC after cardiac surgery in infants and toddlers

Because braces for primary PC are for adolescents without surgical wounds and sternotomy [3–8], infants and toddlers need a different type of brace that must be miniaturized and aligned comfortably for unexpected movement while maintaining an appropriate pressure to prevent damage to wounds and sternum.

**Fig. 1:**
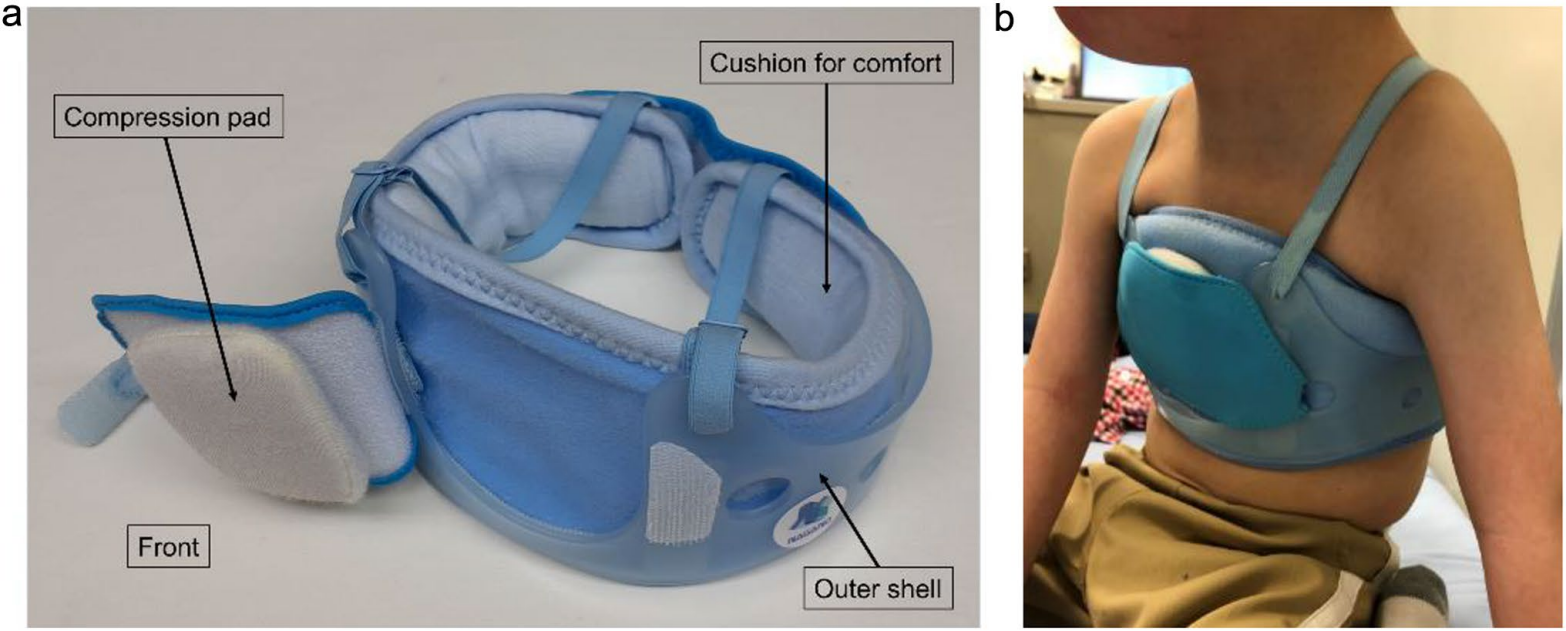
A newly developed brace for infants and toddlers with secondary pectus carinatum. **a** Outer appearance of the brace. **b** View of a patient wearing the brace (4 years old, male, 21-trisomy, 44 months after ventricular septal closure)

Our newly developed brace comprises three main components (Nagano Prosthetics & Orthotics Co., Ltd., Osaka, Japan) (Fig. 1a). First, a lightweight non-deformable outer shell made of polypropylene covers the entire chest without placing excessive stress. Second, a pad connected to the shell with elastic Velcro applies pressure only onto the protruded region through an anterior window created in the shell. Compression pressure was monitored at outpatient visits every 3 months and set at 0.6–0.8 psi (equivalent to 30–40 mmHg) in a sitting position using a portable sensor (Palm Q®, CAPE Co., Ltd., Kanagawa, Japan). The pressure was set for the following reasons to prevent damage to surgical wounds: Human skin capillaries collapse at pressures > 32 mmHg [9]. In addition, a clinical study in wheelchair patients [10] and an in vitro study of wound dressing [11] have shown that compression pressure must be limited to < 40 mmHg to prevent sores with comfort. Third, a lightweight resilient fiber cushion with high breathability (V-Lap®, Teijin Frontier Co., Ltd., Osaka, Japan) was used to cover the inner surface of the brace. The brace was custom-made and renewed when the patient outgrew the current brace (Fig. 1b).

### Measurement of protrusion angle on chest X-ray

We propose a “protrusion angle” measured by lateral chest X-ray (CXR) for objective assessment of the deformity because previous methods assessing the severity of the protrusion in adolescent patients with primary PC, defined by the ratio of the anteroposterior diameter to the traverse diameter of the thorax [3, 4], may not be appropriate for infants and toddlers. This is because the shape of the cross-section of the thorax changes from beer barrel shaped in infants to oblong in adolescence.

**Fig. 2:**
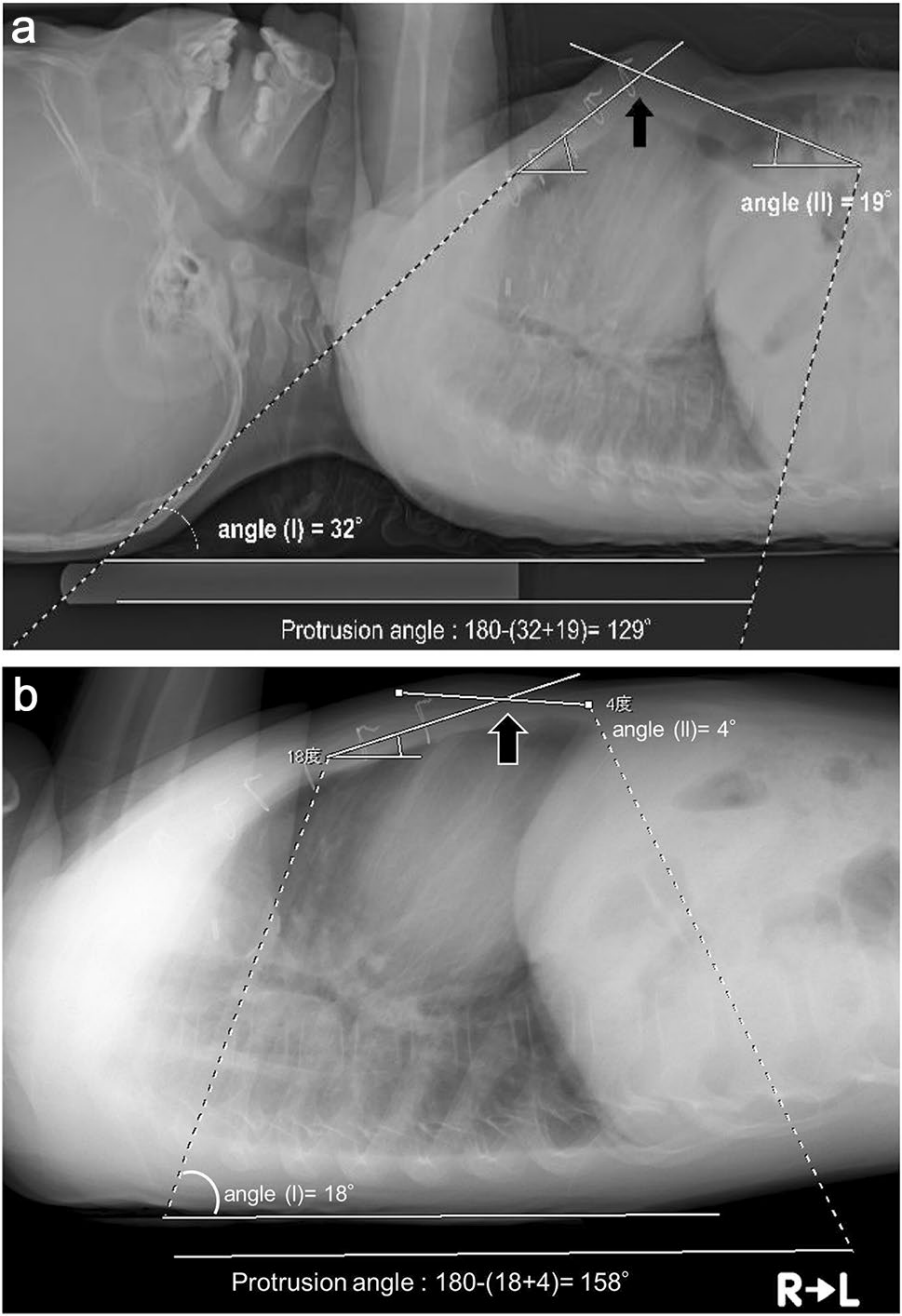
Measurement of the protrusion angle on chest X-ray. The protrusion angle (°) was then defined as 180° minus the sum of angle (I) and angle (II). Angle (I): the angle between the longitudinal axis of the sternum and the dorsal horizontal line of the CXR table. Angle (II): the angle between the descending line from the prominent point of the sternum to the anterior insertion of the diaphragm and the horizontal table line. The solid arrow (➡) indicates the protrusion angle. **a** 129° at the initiation of brace therapy. **b** 158° (+29°) after 66 months of brace therapy (same case as in Fig. 1b)

First, the following angles were measured digitally from the lateral CXR: angle (I) between the longitudinal axis of the sternum and the dorsal horizontal line of the X-ray table and angle (II) between the descending line from the prominent point of the sternum to the anterior insertion of the diaphragm and the dorsal horizontal line. The protrusion angle (°) was then defined as 180° minus the sum of angle (I) and angle (II). The smaller the measurement from 180°, the more severe the protrusion angle. The measurement was repeated at the start of brace compression and periodically Representative measurements of the protrusion angle are shown in Fig. 2a (at the start) and Fig. 2b (after 66 months of brace wearing). To standardize the angle change given under various protrusion severity at the start, the percent change in the angle was calculated as (net change/angle at the start) 100.

### Patient inclusion

There were no exclusion criteria set based on the specific symptoms or physical measurements in this study. When parents claimed a progressive sternal deformity, available treatment options were explained to them. Upon the parents’ request, the patients were referred to our dedicated orthopedic outpatient clinic.

### Brace compression protocol

Actual wear after surgery was started at the time of sternotomy healing, approximately 6 months after surgery. We encourage patients to wear the brace for as long as possible with maximum compliance. Regular outpatient check-ups were performed every 3 months to adjust brace pressure and visual assessment. CXR was performed every 6–12 months to measure the protrusion angle. The brace was renewed when the patient outgrew it.

### Grouping for comparison to extract possible factors affecting changes in the protrusion angle with the brace

We hypothesized that the response of angle change with the brace varied even among patients with the same severity of deformity. To define a cutoff for the borderline, the relationship between severity at the start and extent of percentage change of angle with the brace was estimated by linear regression. The residuals of each patient from the resulting estimates were also calculated. The residuals were set as binary variables that were created as the objective variable of prominent, sparse, or negative percentage change in the angle. For example, a good response to brace therapy was defined as a prominent percentage change when at positive or zero residual for the regression line. The patients were divided into those with positive or zero residuals for the regression line (good responders, Group G) and those with negative residuals (poor responders, Group P).

### Valuables may affect the degree of response to brace compression

Because the pathogenesis of secondary PC focused in this study remains unclear, any possible factors giving mechanical stimulation to the cartilage of the sternum and ribs need to be considered as affecting factors to the angle change besides the patient’s background, i.e., age and body weight at the last surgery, gender, chromosomal abnormality (genetic background), severe cardiac disease (listed in Supplementary table), cardiac enlargement, multiple sternotomy, a complex surgical procedure, delayed chest closure, postoperative severe extracardiac complications, age and body weight at the start of the brace compression, interval between the last surgery and the start of therapy, daily wearing time, total duration of brace wearing, and self-discontinuation.

The complexity of the last surgery was defined as follows: simple surgery—septal defect closure; atrioventricular defect repair; tetralogy of Fallot standard repair; and complex surgery—Rastelli-type operation, arterial switch, one-stage repair for coarctation or interruption of the aorta complex, and Fontan-type operation.

### Statistical analysis

The Wilcoxon/Kruskal–Wallis test and Fisher’s exact test were used to compare ordinal and categorical data between Groups G and P. Univariate and multivariate logistic regression analyses were used to identify factors associated with poor response to brace compression. The results are shown as odds ratio (OR) with 95% confidence intervals (CI).

A value of *p* < 0.05 was used to indicate a significant difference in all analyses. All calculations were performed using JMP Pro 16 (SAS Institute Inc., Cary, NC, USA).

## Results

### Patient pretreatment background

The compression braces were prescribed for 56 children (29 male, 27 female) during the 9-year period between May 2013 and November 2022. Five patients were excluded from the cohort because of loss of follow-up. Therefore, 51 patients were evaluated, where brace therapy was completed in 21 and 7 were still on therapy. The remaining 23 patients discontinued wearing the brace at their own discretion because of unwillingness 16, discomfort in summer 2, immobility 1, entry to elementary school 1, lost follow-up 1, and other 2.

Chromosomal abnormalities were present in 16 patients: 21-trisomy 14 and 22q11.2 deletions 2. Multiple median sternotomies were performed in 24 cases: twice 19, three times 4, and five times 1. The mean age at the last cardiac surgery was 7.7 ± 5.8 months, and the mean age and body weight at the start of brace compression were 14.7 ± 9.2 months and 6.5 ± 2.5 kg, respectively.

The brace was renewed in 8 patients once at a median of 24 months (16–44) postoperatively.

### Grouping

The time course of the protrusion angle and its percentage change in all 51 patients are shown in Supplementary Figs. 1 and 2, respectively. The distribution of the percent change in a histogram and its relationship with the angle at the start of brace compression are shown in Supplementary Figs. 3 and 4, respectively.

**Fig. 3:**
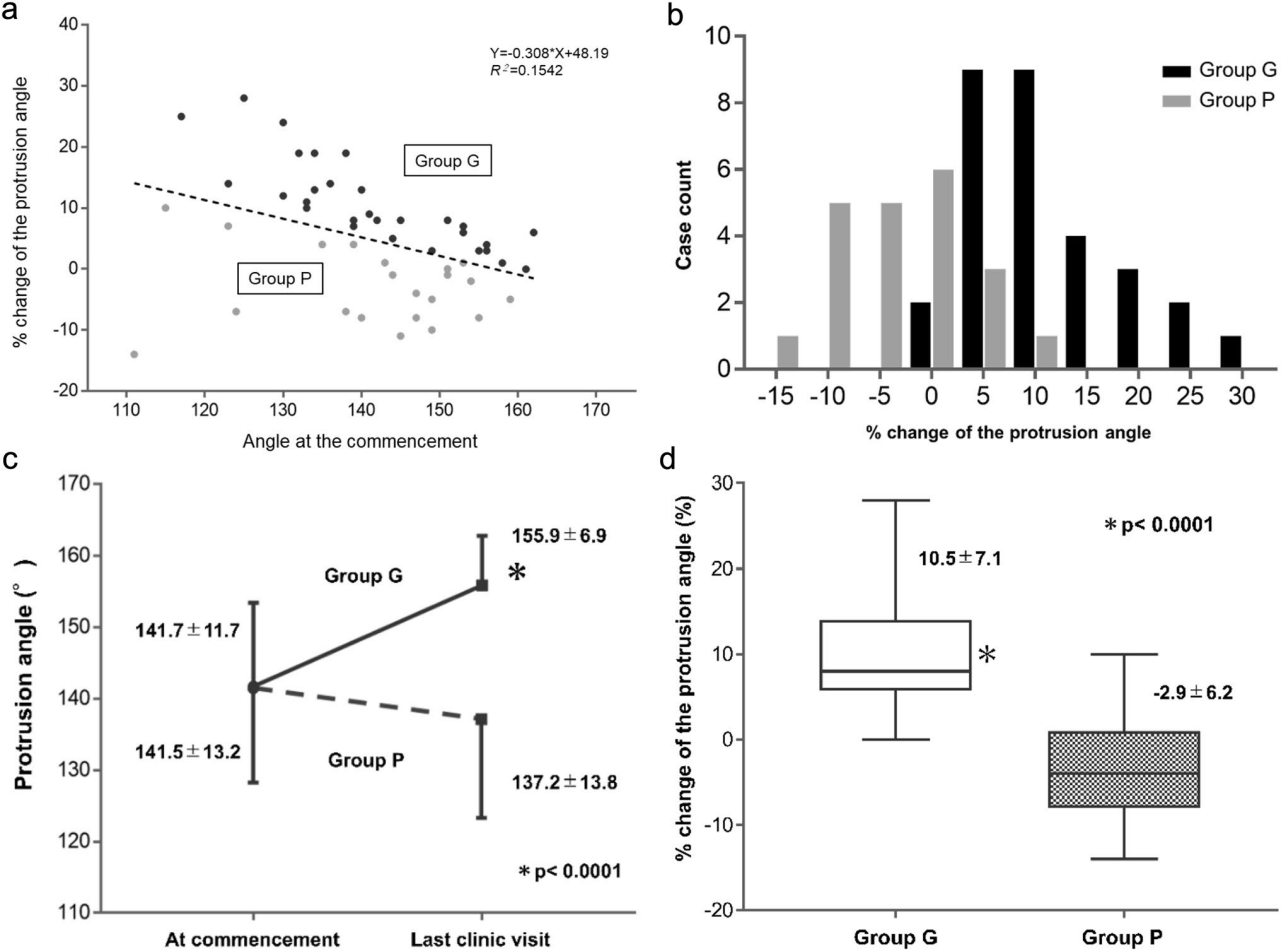
Grouping patients in the relationship between the percentage change of the protrusion angle to the brace compression and the angle at the start and Difference in the response to Brace Compression between Groups G and P. **a** Two groups divided by residuals of the relationship between the final percentage change in the protrusion angle and the angle at the start expressed by a regression line. **b** Distribution of the final percentage change in the protrusion angle of each group shown in a histogram. **c** Comparison of changes in the protrusion angle after brace therapy between the two groups. **d** Comparison of the percentage change in the protrusion angle between the two groups. Group G: Patients with residuals of positive and zero on the regression line, Group P: Patients with residuals of negative to the regression line

According to our proposed grouping method using the regression line (Fig. 3a, *Y* = 48.19 − 0.306*X*, *R*^2^ = 0.15), 30 and 21 cases were divided as in Group G and Group P, respectively. As a result, 58.8% (30/51 patients) responded well to brace compression. Histograms of the percentage change in the two groups are shown in Fig. 3b.

There was no significant difference in the protrusion angle at the start between the two groups, but it was significantly smaller in Group G with brace than in Group P (155.9 ± 6.9° vs. 137.2 ± 13.8°, *p* < 0.0001, Fig. 3c). The percentage change in the angle was significantly greater in Group G than in Group P (10.5 ± 7.1° vs. −2.9 ± 6.2°, *p* < 0.0001, Fig. 3d).

### Comparison between Groups G and P

Table 1 presents the patient background of each group at the start of the study. The proportion of males, severe cardiac anomaly, multiple median sternotomy, and complex procedure of the last surgery were significantly greater in Group P.

**Table 1:** Pretreatment patient background

Variables	All (*n* = 51)	Group G (*n* = 30)	Group P (*n* = 21)	*p*
Age at surgery (months)	7.7 ± 5.8	6.5 ± 4.2	9.3 ± 7.4	0.125
Body weight (kg)	6.5 ± 2.5	6.4 ± 2.6	6.7 ± 2.4	0.379
CTR (%)	53.6 ± 5.3	55.3 ± 5.8	57.5 ± 5.9	0.150
Gender (male/female)	25/26	11/19	14/7	0.048
Chromosomal abnormality (yes/no)	14/37	9/21	5/16	0.754
Severity of cardiac abnormality (mild/severe)	38/13	26/4	12/9	0.024
Median sternotomy (single/multi)	27/24	20/10	7/14	0.025
Complexity of the last surgery (simple/complex)	35/16	25/5	10/11	0.013
Delayed chest closure (yes/no)	5/46	2/30	3/21	0.637
Complications including respiratory, infectious, and developmental events (yes/no)	38/13	22/8	16/5	1

Data in parenthesis are SD. Categorical variables are shown as case counts

The status of brace compression is listed in Table 2. There was no significant difference in age at the start and in the interval from the last surgery to the start between the two groups. While daily wearing time insignificantly differs between the two groups (Group G 6.6 ± 3.6 vs. Group P 6.2 ± 4.9 h/day, *p* = 0.658), the total duration of brace compression was significantly shorter in Group P than in Group G (34.4 ± 14.2 vs. 45.3 ± 19.3 months, *p* = 0.046).

**Table 2:** Status of the brace therapy

Variables	All (*n* = 51)	Group G (*n* = 30)	Group P (*n* = 21)	*p*
Age at start of brace therapy (months)	14.7 ± 9.2	13.4 ± 7.7	16.5 ± 10.9	0.367
Interval between last surgery and start of brace therapy (months)	6.6 ± 7.4	6.2 ± 6.9	7.2 ± 8.2	0.494
Daily wearing time (hours)	6.4 ± 4.2	6.6 ± 3.6	6.2 ± 4.9	0.658
Total duration of brace therapy (months)	40.8 ± 18.0	45.3 ± 19.3	34.4 ± 14.2	0.046
Completed/On-going/Self-discontinuation	21/7/23	16/5/9	5/2/14	0.045

Data in parenthesis are SD. Categorical variables are shown as case counts

### Factors influencing response to brace compression

As shown in Table 3, univariate analysis showed that male gender, severe cardiac anomalies, multiple median sternotomy, complex surgical procedures, shorter duration of brace therapy, and self-discontinuation were significantly associated with poor response to brace compression. On the other hand, multivariate analysis showed that male gender, severe cardiac anomaly, and complex surgical procedure, although not significant, remained factors with a high odds ratio for poor response.

**Table 3:** Factors influencing therapeutic response to compression brace

Variables	Univariate			Multivariate		
	OR	95% CI	*P*	OR	95%CI	*P*
Patient background variables						
Age at last surgery (1 month increment)	1.10	0.98–1.25	0.11			
Gender (male)	3.45	1.09–11.7	0.03	2.8	0.99–1.09	0.12
Chromosomal abnormality (+)	0.73	0.19–2.55	0.63			
Severe cardiac abnormalities	4.87	1.31–21.09	0.017	2.2	0.72–11.11	0.595
CTR (1% increment)	1.07	0.97–1.19	0.19			
Surgery-relating variables						
Median sternotomy (multiple)	4.0	1.26–13.7	0.018	1.8	0.33–9.54	0.496
Complex surgical procedure (last surgery)	5.5	1.59–21.52	0.0067	6.7	0.46–99.58	0.165
Delayed chest closure (+)	2.3	0.35–15.36	0.38			
Severe complications	1.16	0.32–4.47	0.82			
Brace-relating variables						
Age at start (1 month increment)	1.04	0.98–1.11	0.24			
Body weight at start (1 kg increment)	1.07	0.85–1.35	0.58			
Interval between the last surgery and commencement (1 month increment)	1.02	0.94–1.10	0.64			
Daily wearing time (1 h increment)	0.98	0.85–1.12	0.76			
Total duration (1 month increment)	0.96	0.92–1.00	0.04	1.03	0.99–1.09	0.12
Self-discontinuation (vs. completed and ongoing)	4.67	1.41–15.45	0.012	1.9	0.99–1.09	0.41

OR, odds ratio; CI, confidence intervals

There were no adverse events such as pain, wound damage, or sternum dehiscence throughout the observation period.

## Discussion

Three major findings were obtained in this study. First, using our new direct measurement of anterior protrusion angulation, patients were reasonably divided into two comparable groups in a nonarbitrary manner, using the calculated residuals to the relationship between the severity of the protrusion at the start and the percentage of protrusion angle by compression. Second, while positive angle change was acknowledged in 68.6% (35/51) of patients, good response to the brace fell at a rate of 58.8% (30/51 patients) by our definition. Third, comparisons between these two different response groups enabled us to extract factors with a high odds ratio affecting poor response to brace compression.

In primary PC, definitions or criteria of therapeutic improvement by brace compression have been unclear in the previous reports because of indirect measurement of the anterior protrusion with subjective visual impression of the chest deformity [5–7] or with ratio of the length and width of the thorax [3, 4]. Instead, we performed a direct measurement of the protrusion itself as an angle to express severity in a simple, reproducible, and precise manner. Because the protrusion is the primary focus of brace compression, the angle is the only direct reflection of the treatment response to the brace. An ideal study protocol should include two comparative groups with or without a target therapy to assess the effectiveness of a new therapy and its affecting factors. However, with several limitations mentioned in the Methods section, it was necessary to construct a new validation method that would allow comparison in an one-way study. We proposed a new approach to create two comparison groups using a relationship between the severity of the protrusion at the start and the degree of angle change with the brace. As one can predict, when using another clinical factor as an independent parameter instead of or combined with protrusion severity, the patients that comprise the groups may change.

The rate of good response to our current brace protocol was relatively low (58.8%) according to our proposed criteria, whereas the success rate in primary PC was reported as more than 70% [6–8]. There are two possible reasons for the different success rates between primary and secondary PCs. First, the target compression pressure in this study may have been low and unstable. The compression pressure in the primary PC was set at a pressure that bent the sternum to a visible extent [5–7], which was five to ten times greater than that of our brace. Outpatient visits every 3 months may have not been frequent enough to maintain the compression pressure within the target range. Second, the total duration of brace compression was another significant factor affecting the response because significantly less angle change was observed in Group P with a higher rate of patients who self-discontinued brace wearing. This finding confirms the importance of compliance even in the brace for the secondary PC, as emphasized in the primary PC for a successful outcome [5–7, 12]. On the other hand, given that the patients with good angle changes had been wearing the brace for four years, we must not only encourage the patients for better compliance but also improve the protocol to be effect in a short time along with better brace performance.

Many surgeons examining patients with secondary PC agree with the extracted worsening factors causing mechanical instability to the sternum– rib cartilage that may provoke proliferative remodeling leading to deformity and thickening. Thus, the prophylactic use of the brace could be considered even earlier than 6 months postoperatively or in the interval between staged surgeries.

Although a compression brace could be overall acceptable for secondary PC after cardiac surgery, one might need to consider surgical treatment established well in primary PC [7, 13]. However, anatomical or procedural constraints may limit surgical options in infants with secondary PC.

## Limitations

This study had several unavoidable limitations as a retrospective one-way cohort. This was a single-center retrospective study with a relatively limited follow-up period. Second, this study was conducted without a control group or randomization, which is the mainstay for directly defining the efficacy of a novel therapy. Finally, the precise pathogenesis of cartilage deformity and proliferation in secondary PC remains unclear, which could be the primary target of any treatment or prevention.

## Conclusions

Our newly developed compression brace contributed, at least in part, to reduce the anterior chest protrusion in secondary PC in infants and toddlers undergoing cardiac surgery. The findings of this study can be confirmed by clarifying the natural history of this secondary PC or by performing a comparative study with and without the braces. We expect to bring this small but important unsolved issue to the surgical community for attention.

## Supplementary Information

Below is the link to the electronic supplementary material.

Supplementary file1 (DOCX 24 KB)

Supplementary file2 (PDF 314 KB)

## Data Availability

All relevant data are within the manuscript. The data underlying this study will be shared upon reasonable request by the corresponding author.
