# Supplementary material for: Compression brace for secondary pectus carinatum in infants and toddlers undergoing cardiac surgery with midline sternotomy
Source: Gen Thorac Cardiovasc Surg. 2024 Apr 25;72(11):718–25. doi: 10.1007/s11748-024-02030-0 (PMC11471696; doi:10.1007/s11748-024-02030-0)
Supplement: Supplementary file 1 — Supplementary file1 (DOCX 24 KB) [file 11748_2024_2030_MOESM1_ESM.docx]

**Supplemental table**

| Cardiac diagnosis |  | All (n=51) | Group G (n=30) | Group P (n=21) |
| --- | --- | --- | --- | --- |
| Ventricular septal defect |  | 21 | 18 | 3 |
| Atrial septal defect |  | 2 | 2 |  |
| Atrioventricular septal defect |  | 4 | 1 | 3 |
| Tetralogy of Fallot |  | 6 | 3 | 3 |
| Double outlet right ventricle |  | 2 |  | 2 |
| Pulmonary atresia/Ventricular septal defect | | 3 | 1 | 2 |
| Transposition of the great arteries | | 4 | 1 | 3 |
| Single ventricle |  | 3 | 1 | 2 |
| Total anomalous pulmonary venous drainage | | 2 | 1 | 1 |
| Coarctation of the aorta |  | 1 |  | 1 |
| Truncus arteriosus |  | 1 |  | 1 |
| Pulmonary stenosis |  | 1 | 1 |  |
| Interruption of the aorta |  | 1 | 1 |  |
