# Supplementary material for: Compression brace for secondary pectus carinatum in infants and toddlers undergoing cardiac surgery with midline sternotomy
Source: Gen Thorac Cardiovasc Surg. 2024 Apr 25;72(11):718–25. doi: 10.1007/s11748-024-02030-0 (PMC11471696; doi:10.1007/s11748-024-02030-0)

Supplementary Fig. 1

Response to brace compression：Time course of the protrusion angle throughout brace therapy in all patients

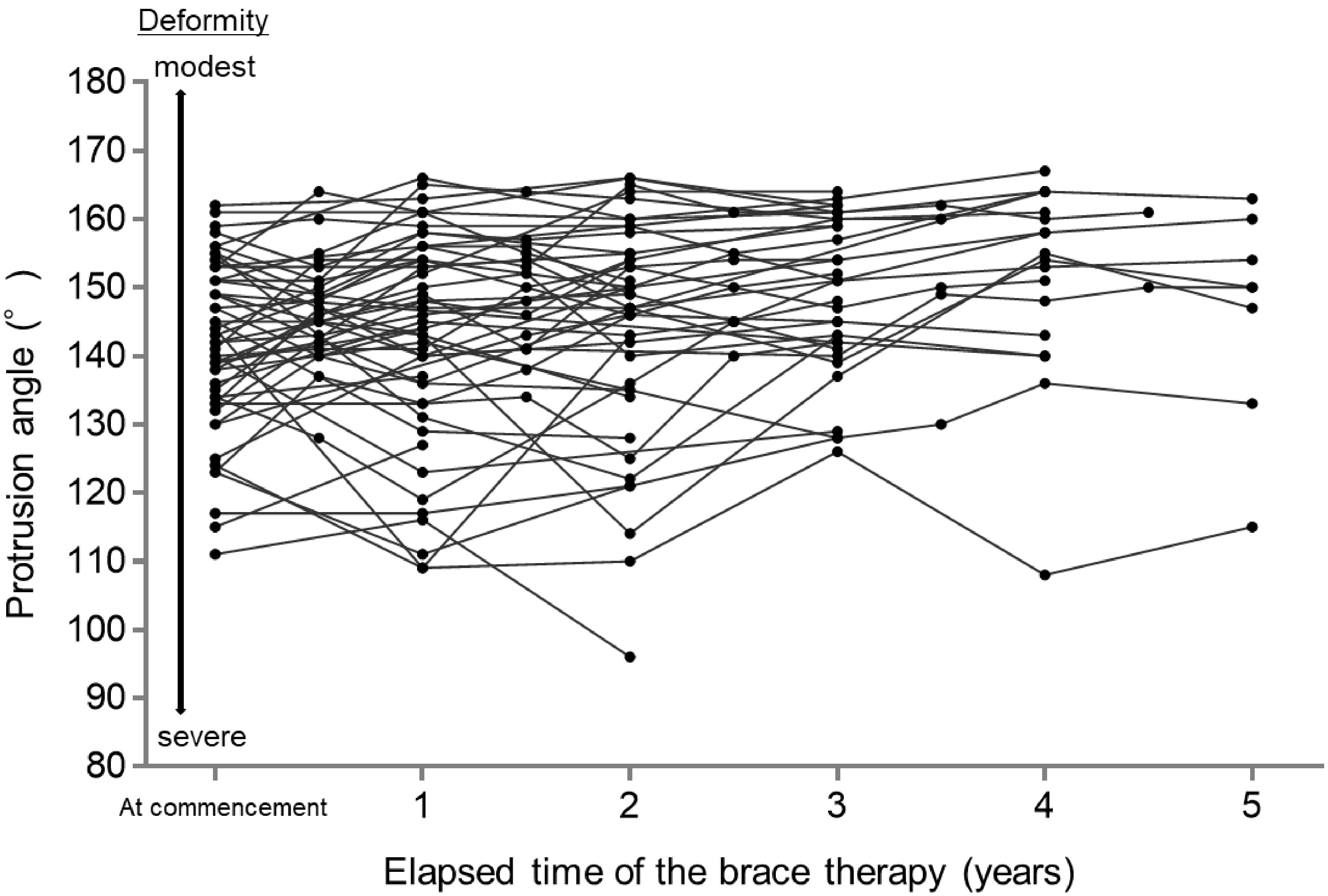

Supplementary Fig. 2

Response to brace compression: Time course of percentage change in the angle throughout brace therapy in all patients

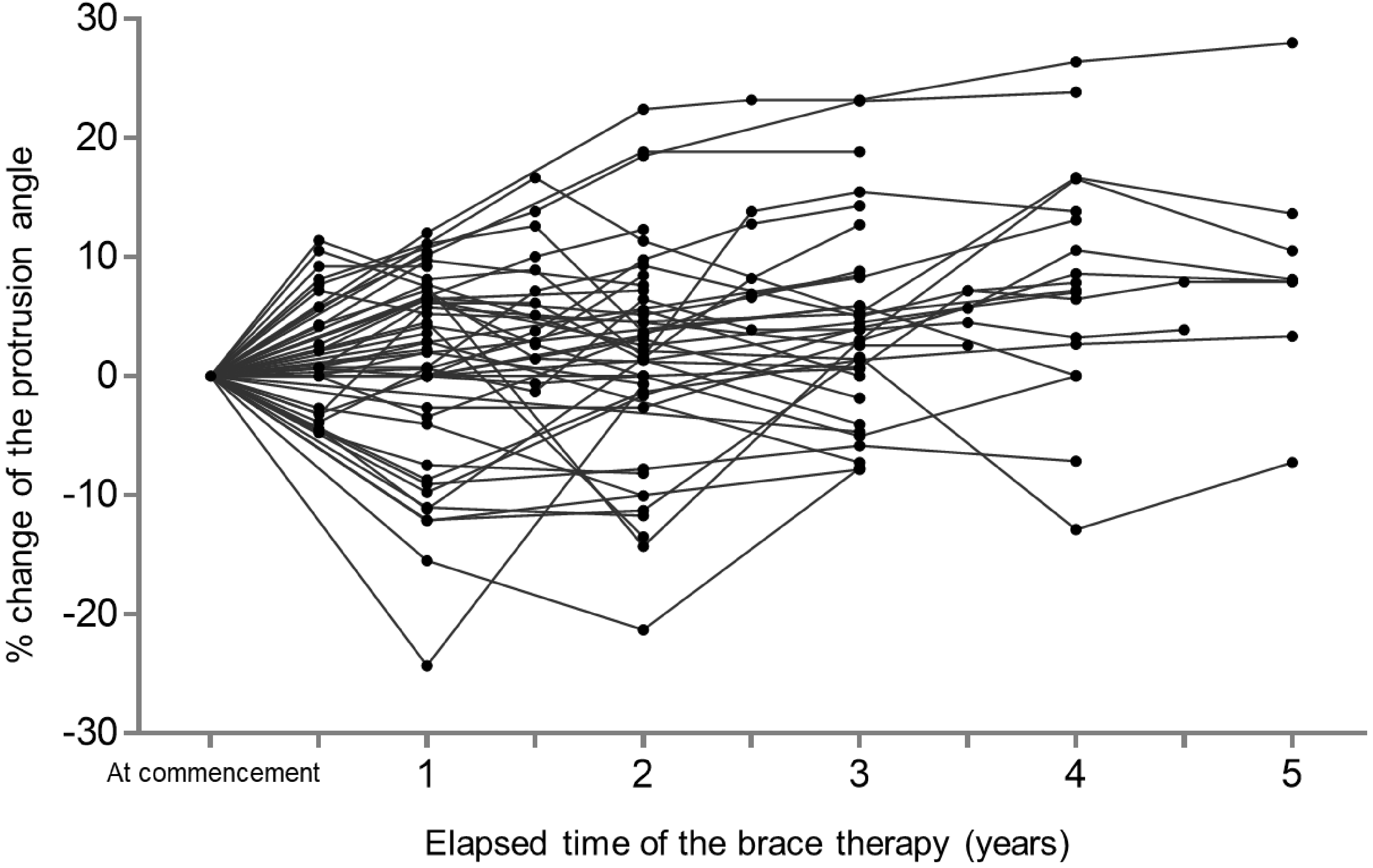

Supplementary Fig. 3

Response to brace compression: Distribution of percentage change in the angle at the final clinic visit expressed in a histogram

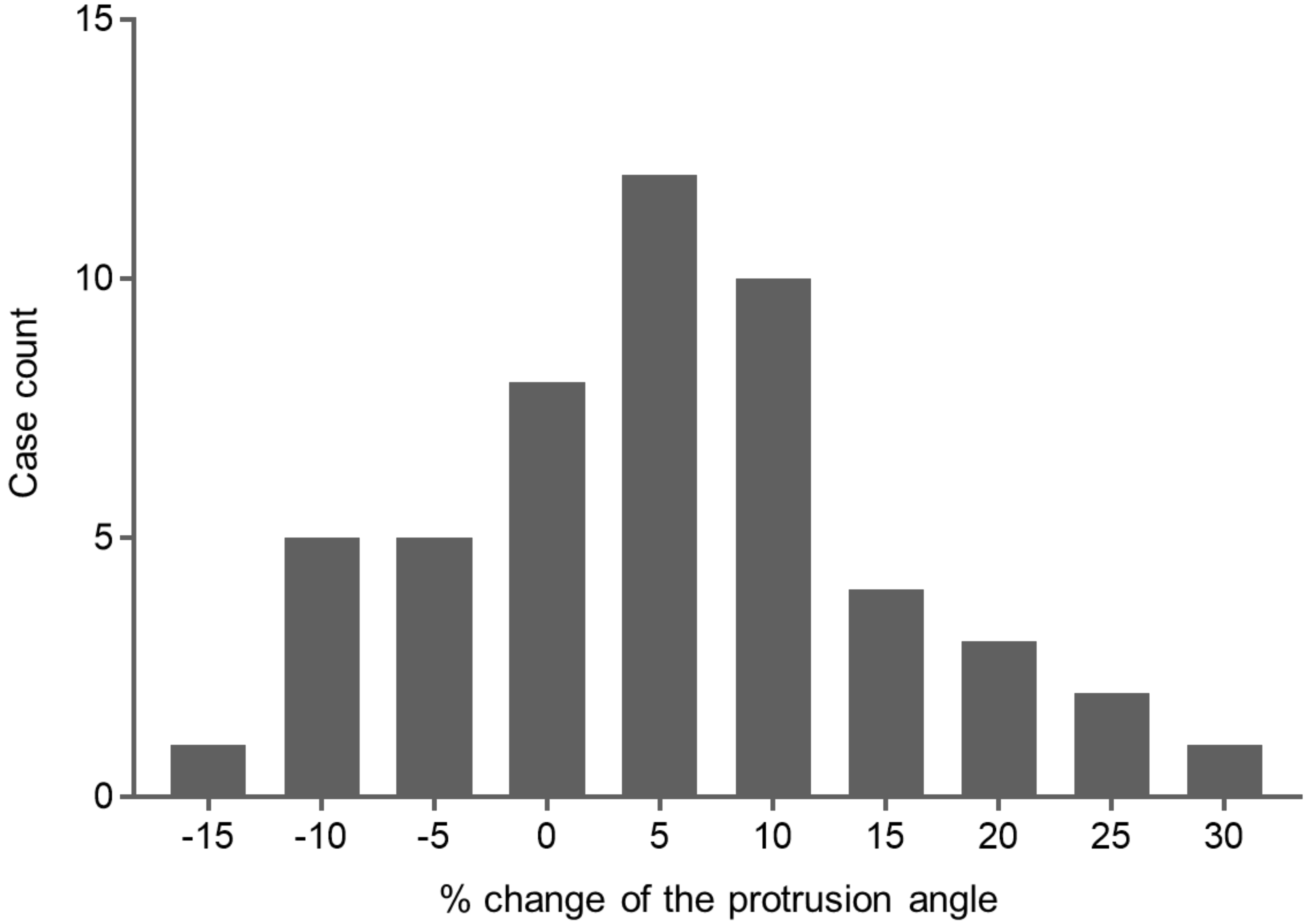

Supplementary Fig. 4

Response to brace compression: Distribution of percentage change in the angle at the final clinic visit in relation to the protrusion angle at the initiation of brace therapy in all patients

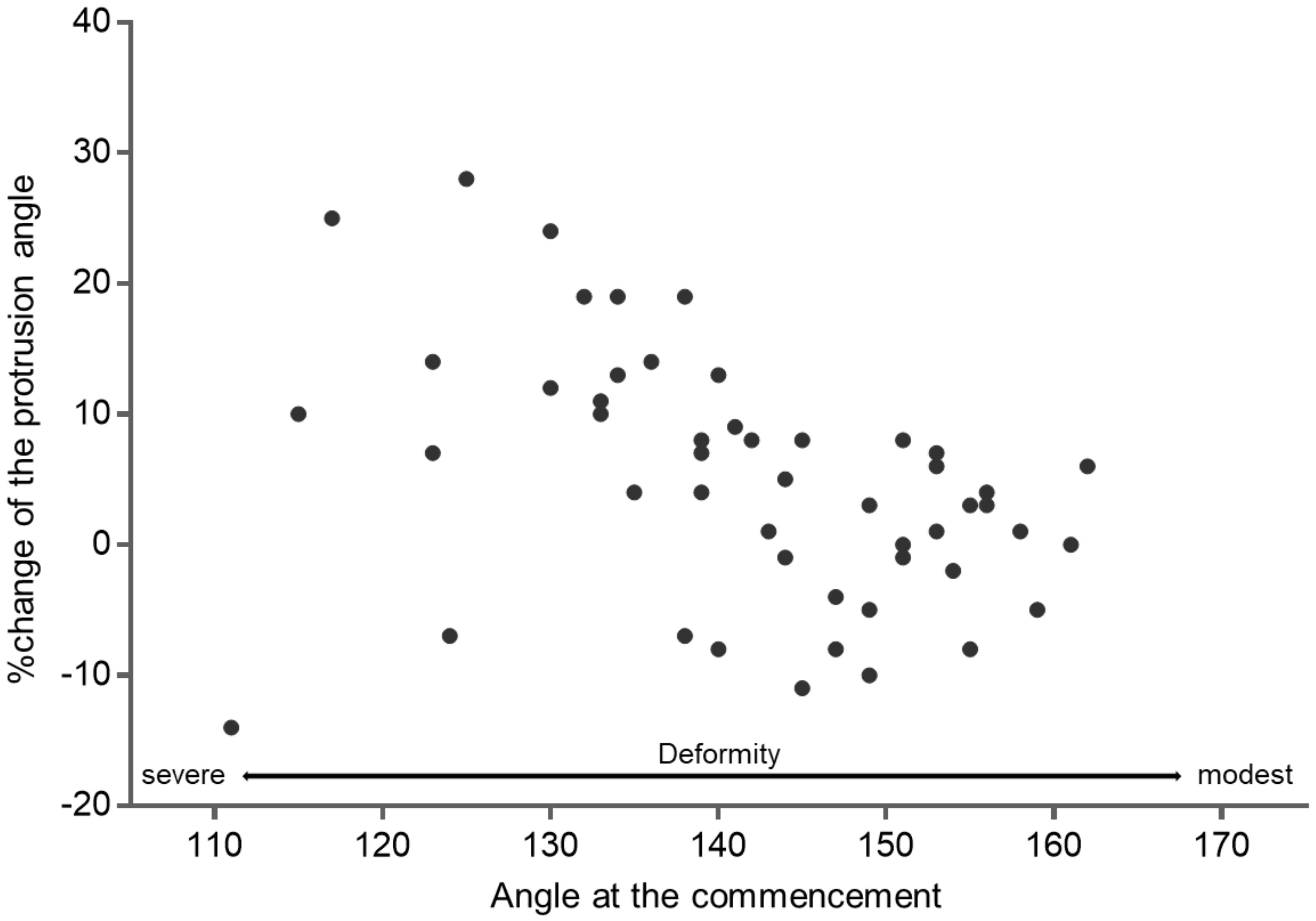

Supplement: Supplementary file 2 — Supplementary file2 (PDF 314 KB) [file 11748_2024_2030_MOESM2_ESM.pdf]
